# Combining Silica-Loaded Iron-Catalyzed Sodium Percarbonate (SPC^SF^) with *Bacillus subtilis* for Enhanced Remediation of Diesel-Contaminated Soil: Performance and Synergistic Mechanisms

**DOI:** 10.3390/ma19122510

**Published:** 2026-06-10

**Authors:** Beibei Ren, Wei Wei, Mingli Wei, Guangsi Zhao

**Affiliations:** 1 State Key Laboratory of Intelligent Construction and Healthy Operation and Maintenance of Deep Underground Engineering, China University of Mining and Technology, Xuzhou 221116, China; renbeibei@jsviat.edu.cn; 2 School of Transportation Engineering, Jiangsu University of Architectural Technology, Xuzhou 221116, China; 3 School of Mechanics and Civil Engineering, China University of Mining and Technology, Xuzhou 221116, China; 4 State Key Laboratory of Geomechanics and Geotechnical Engineering, Institute of Rock and Soil Mechanics, Chinese Academy of Sciences, Wuhan 430071, China; weimingli830716@sina.com; 5 Jiangsu Institute of Zoneco Co., Ltd., Yixing 214200, China

**Keywords:** Fenton-like oxidation, microbial remediation, hydrocarbon pollution, coupled remediation

## Abstract

Petroleum hydrocarbons contamination in soil is difficult to remediate due to strong adsorption and limited bioavailability. This study investigated the coupled remediation of diesel contamination in an alkaline kaolin-based model substrate using a silica gel-loaded, iron-catalyzed sodium percarbonate composite (SPC^SF^) and *Bacillus subtilis*. The alkaline model substrate was used as a simplified representation of difficult-to-reclaim hydrocarbon- and reagent-impacted matrices that may occur at oil drilling or production sites. In this study, a combined remediation strategy integrating a silica gel-loaded, iron-catalyzed sodium percarbonate composite (SPC^SF^) with *Bacillus subtilis* ATCC 11774 was developed for diesel-contaminated soil. The remediation performance of chemical oxidation, microbial remediation, and their combined application was systematically evaluated. The simultaneous SPC^SF^–microbial treatment achieved the highest removal efficiency, reaching 65.1% after 31 d, which was markedly higher than that of chemical oxidation (22.5%) or microbial remediation alone (31.1%). Within the mineral model substrate used in this study, SPC^SF^ effectively regulated pH and oxidation–reduction potential, creating conditions more favorable for microbial activity. Spectroscopic analyses (three-dimensional fluorescence spectrum, Fourier transform infrared spectroscopy, and X-ray photoelectron spectroscopy) indicated that SPC^SF^ promoted the transformation of diesel hydrocarbons into bioavailable intermediates, which were further converted by microorganisms into carboxyl-rich organic matter. *Bacillus subtilis* was associated with a higher Fe(II) proportion in the coupled system, which may have favored maintenance of Fe redox activity and sustained Fenton-like reactivity. However, direct measurements of reactive oxygen species and Fe(II)/Fe(III) dynamics were not performed; therefore, this interpretation should be regarded as a plausible hypothesis based on indirect evidence. The specific microbial contribution to Fe redox transformation was inferred from indirect evidence and may also have been influenced by medium-derived components or microbial metabolites. This study presents a coupled supported sodium percarbonate and microbial remediation strategy providing mechanistic evidence for the compatibility of supported chemical oxidation and microbial degradation in diesel-contaminated soil.

## 1. Introduction

Petroleum hydrocarbons, as essential resources for industrialization, have been extensively exploited worldwide [1,2]. Increasing demand has driven the construction of numerous petroleum extraction and storage facilities (e.g., oil wells and storage tanks), including oil wells and storage tanks, which has consequently led to frequent leakage events and transportation accidents [3,4]. These incidents cause severe ecological damage, manifested by soil fertility degradation and alterations in soil physicochemical properties. As a result, petroleum pollution has emerged as a global environmental concern [5]. Current remediation technologies are generally classified into physical, chemical, and biological approaches [6,7,8,9]. Among them, microbial remediation has attracted considerable attention because it produces no secondary pollution, is cost-effective, and exhibits strong sustainability [7]. However, its effectiveness is often limited in degrading structurally complex petroleum hydrocarbons that are strongly adsorbed onto soil particle surfaces. Moreover, remediation performance is highly sensitive to environmental conditions such as temperature and soil pH [10,11], and the process typically requires long treatment periods, resulting in substantial time costs.

To address these limitations, chemical oxidation has recently been integrated with microbial remediation [8,12]. Chemical oxidation can rapidly degrade recalcitrant compounds; however, it generally requires large quantities of oxidants. Excessive oxidant dosages have been reported to adversely affect soil properties, including pH and organic matter content, thereby impairing the soil environment [13]. By contrast, the combined strategy mitigates the low efficiency of microbial remediation and reduces the high oxidant demand and associated environmental risks of chemical oxidant, making it a promising approach for soil remediation. In addition to chemical oxidation and bioremediation, adsorbent materials have also been investigated for petroleum product removal from contaminated media. Such materials can effectively capture or immobilize hydrocarbon pollutants and may serve as a useful option for petroleum-contaminated substrates [14]. However, adsorption mainly transfers or immobilizes contaminants rather than promoting their chemical transformation or biodegradation. Therefore, the present study focuses on oxidation–biodegradation coupling as a strategy aimed at both pollutant activation and subsequent microbial transformation.

Despite these advantages, many commonly used oxidants exhibit pronounced microbial toxicity, which restricts their applicability in combined remediation systems [15,16]. For instance, sodium persulfate generates SO_4_^−^· radicals that can inhibit or inactivate microorganisms [17,18]. Although the Fenton reagent is less toxic than persulfate, high concentrations still significantly suppress microbial activity [19]. Even though microbial communities may partially recover after treatment, excessive oxidant exposure can induce irreversible changes in community structure and genetic composition [20]. In addition, the short half-life of H_2_O_2_ (from minutes to hours) poses a further challenge. Nonuniform distribution in soil can create localized regions with high H_2_O_2_ concentrations, which promotes direct decomposition into H_2_O and O_2_ rather than participation in Fenton reactions [21]. This competing pathway dominates H_2_O_2_ consumption and markedly reduces oxidation efficiency.

Sodium percarbonate (SPC) has recently been recognized as a promising oxidant for organic contamination remediation [22,23]. As an H_2_O_2_-Na_2_CO_3_ adduct, SPC decomposes in aqueous environments to release H_2_O_2_, Na^+^, and CO_3_^2−^, thereby enabling pollutant degradation through Fenton reactions. In comparison with direct H_2_O_2_ application, SPC offers improved handling safety, enhanced transportation security, and broader pH adaptability. Its slow-release behavior effectively prevents localized accumulation of H_2_O_2_, which reduces microbial toxicity and undesired side reactions while improving H_2_O_2_ utilization efficiency. Furthermore, Pan, et al. [24] demonstrated that SPC addition can stimulate microbial metabolic activity in contaminated sediments by supplying electron acceptors or donors, highlighting its potential as a cost-effective ecological engineering strategy. In previous work, we further improved component stability and remediation efficiency by developing silica-based carriers for SPC and FeSO_4_ in Fenton systems.

Evaluating biodegradation in petroleum hydrocarbon-contaminated soil requires both macroscopic removal data and advanced analytical evidence capable of tracking the transformation of organic intermediates, surface functional groups, and redox-active species during remediation. Three-dimensional excitation-emission matrix fluorescence spectroscopy (3D-EEM), Fourier transform infrared spectroscopy (FTIR), and X-ray photoelectron spectroscopy (XPS) provide complementary information on dissolved organic matter evolution, carbon functional group transformation, and Fe/C chemical-state variation, respectively. These techniques effectively distinguish whether pollutant removal is driven by the microbial conversion of oxidation products into more biodegradable forms, rather than solely by abiotic oxidation. While existing studies on coupled oxidation–bioremediation systems primarily emphasize overall removal efficiency, spectroscopic and redox-scale evidence for biodegradation remains limited. Therefore, this study combines performance evaluation with 3D-EEM, FTIR, and XPS analyses to provide a mechanistic understanding of biodegradation behavior in the SPC^SF^–microbial remediation system.

In this study, *Bacillus subtilis* was combined with a silica gel-loaded, iron-catalyzed SPC composite (SPC^SF^) for the remediation of diesel-contaminated soil. This strain was selected as a representative aerobic hydrocarbon degrader because *Bacillus* spp. are known for their environmental robustness and their ability to transform diesel-range petroleum hydrocarbons. Diesel-range petroleum hydrocarbons are a complex mixture mainly composed of n-alkanes, branched alkanes, cycloalkanes, and aromatic hydrocarbons. Among these constituents, the aliphatic fraction constitutes a major proportion of diesel and was therefore selected in this study as the primary analytical target for comparing remediation performance. However, in the present study, the analytical focus was placed primarily on the diesel-range aliphatic fraction because it constitutes a major and readily quantifiable component of diesel and provides a practical indicator for comparing the relative performance of oxidation, biodegradation, and coupled remediation treatments. This scope should not be interpreted as implying that aromatic hydrocarbons are unimportant; rather, compound-specific analysis of aromatic fractions such as BTEX and PAHs was beyond the scope of the present work and should be included in future studies for a more comprehensive assessment of environmental risk reduction. Unlike previous studies focusing on standalone chemical oxidation, standalone biodegradation, or loosely coupled systems, this work develops a supported SPC-based oxidation system with improved compatibility toward microbial remediation and systematically evaluates its synergistic mechanisms. The remediation performance of SPC^SF^ alone, microbial treatment alone, and two combined modes (sequential and simultaneous) were compared to identify the optimal strategy. The effect of SPC^SF^ on the soil physicochemical environment, particularly pH and oxidation–reduction potential (ORP), was evaluated to determine how the oxidant system regulates microbial activity. The remediation mechanisms of the SPC^SF^–bacteria system were analyzed using XPS, FTIR, and 3D-EEM. These findings demonstrate the feasibility of coupling supported sodium percarbonate-driven chemical oxidation with microbial degradation and provide molecular- and redox-related evidence for diesel transformation. The results also suggest the possible involvement of Fe redox transformation and microbial contributions to the stabilization of the coupled remediation system, although the specific linkages among microbial activity, Fe(II)/Fe(III) cycling, and sustained oxidizing reactivity remain hypothetical within the present experimental framework. The main contributions of this study are the development of a supported SPC–microbial coupled remediation system, the identification of the optimal coupling mode, and the clarification of the regulatory role of the supported oxidant system in the physicochemical environment of the kaolin-based model substrate used in this study.

The present work focuses on mechanistic evaluation under a representative SPC^SF^ dosage, whereas systematic dosage optimization for engineering deployment remains beyond the scope of this study.

## 2. Materials and Methods

### 2.1. Soil and Reagent

The test soil used in this study was commercial kaolin. As one of the fundamental clay minerals widely present in natural soils, kaolinite is commonly used as a model soil in laboratory experiments. In addition, commercial kaolin contains a low organic matter content, which helps to minimize interference from background organic substances with reaction processes. Therefore, the use of kaolin in this study was intended to provide a simplified and well-controlled matrix for identifying the coupled effects of SPC^SF^ and *Bacillus subtilis* on diesel transformation, Fe speciation evolution, and physicochemical regulation. Commercial 0# diesel, a representative diesel hydrocarbon, was selected as the target contaminant. The physicochemical properties of the kaolin and 0# diesel are presented in Table 1. The relatively high initial pH of the contaminated matrix was primarily associated with the intrinsic alkalinity of the commercial kaolin rather than the diesel itself. Thus, the initial pH condition reflected the properties of the selected model soil matrix.

However, it should be noted that this simplified matrix does not fully represent the complexity of natural soils. In real field soils, background organic matter, competing inorganic ions, mineral heterogeneity, and native microbial communities may substantially influence oxidant consumption, radical utilization efficiency, microbial competition, and contaminant accessibility. Therefore, the results obtained here should be interpreted primarily as mechanistic evidence under controlled conditions, and further validation in natural soils is needed before field-scale application.

**Figure 1:**
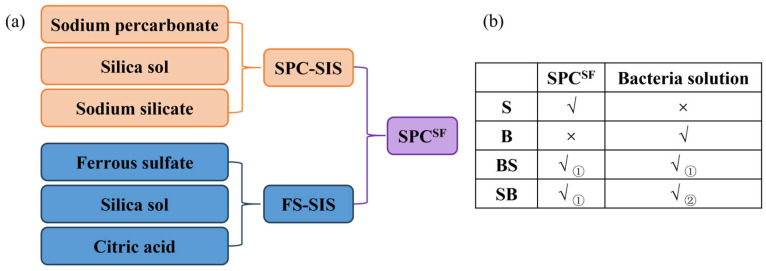
Schematic diagram of SPC^SF^ preparation and experimental design. In (**a**), SPC-SIS was used as the oxidizing agent and FS-SIS was used as the catalyst. (**b**) illustrates the general treatment framework, including oxidation alone, microbial remediation alone, sequential combined remediation, and simultaneous combined remediation. The specific dosage combinations of SPC^SF^ (1% and 3% of dry soil weight) and bacterial suspension volumes are provided in Table 2. In (**b**), “√” indicates that the corresponding remediation approach was applied, whereas “×” indicates that it was not applied. Symbols ① and ② denote the sequence of remediation application. “S” denotes SPC^SF^ dosage (%), and “B” denotes the volume (mL) of *Bacillus subtilis* suspension added to 100 g of dry soil. Codes in the form of BxSy represent simultaneous combined treatments, whereas codes in the form of SxBy represent sequential combined treatments.

Chemical oxidation was conducted using supported sodium percarbonate as the oxidizing agent and supported ferrous sulfate as the catalytic material. The composition of the supported sodium percarbonate and supported ferrous sulfate are summarized in Figure 1a.

The diesel hydrocarbon-degrading bacterium used in this study was *Bacillus subtilis* ATCC 11774, which has been reported to exhibit effective hydrocarbon degradation capability. This strain was selected because *Bacillus* spp. are widely recognized as environmentally robust hydrocarbon degraders capable of utilizing diesel-range hydrocarbons and related aliphatic compounds as carbon sources. According to previous studies [31], *Bacillus subtilis* generally performs well under neutral to mildly alkaline conditions, whereas strongly alkaline environments are not considered optimal for microbial growth and biodegradation activity. The strain was purchased from China Biosep Group Co., Ltd., Xi’an, China.

**Table 2:** Experimental treatment groups and dosage settings of SPC^SF^ and bacterial suspension.

Group	Remediation Mode	SPC^SF^ (%)	Bacteria Solution (mL)	Inoculation Timing
Blank	Sterile control	0	0	/
S1	SPC^SF^ only	1	0	Day 0
S3	SPC^SF^ only	3	0	Day 0
B10	Bacteria only	0	10	Day 0
B20	Bacteria only	0	20	Day 0
B30	Bacteria only	0	30	Day 0
B10S1	Simultaneous combined	1	10	Day 0
B20S1	Simultaneous combined	1	20	Day 0
B30S1	Simultaneous combined	1	30	Day 0
B30S3	Simultaneous combined	3	30	Day 0
S1B10	Sequential combined	1	10	Day 10
S1B20	Sequential combined	1	20	Day 10
S1B30	Sequential combined	1	30	Day 10
S3B30	Sequential combined	3	30	Day 10

Note: “B” denotes the volume (mL) of the *Bacillus subtilis* suspension added to 100 g of dry contaminated soil, while “S” denotes the dosage (%) of SPCSF. For example, B30S1 indicates simultaneous treatment with 30 mL bacterial suspension and 1% SPCSF, whereas S1B30 indicates sequential treatment with 1% SPCSF followed by 30 mL bacterial suspension.

### 2.2. Artificial Contamination

The kaolin was passed through a 2 mm sieve, and the soil samples were oven-dried at 105 °C for 24 h, followed by cooling to room temperature and sealed storage. Diesel was dispensed onto the kaolin surface in small aliquots at multiple locations using a pipette, followed by thorough manual mixing for 5 min to achieve a homogeneous contaminant distribution. The contaminated soil was then aged at 4 °C for 7 days. The initial petroleum hydrocarbon concentration in the kaolin was 10,229.640 mg/kg. Because diesel was introduced as the contaminant rather than as a pH-regulating agent, the strongly alkaline initial condition of the contaminated soil was primarily inherited from the kaolin matrix.

### 2.3. Preparation of Oxidizing Agents and Microbial Cultivation

The preparation procedure of the chemical oxidation agents followed a previously reported method [32]. *Bacillus subtilis* ATCC 11774, a strain reported to possess petroleum hydrocarbon degradation ability, was used in this study. The strain was purchased from Baolin Plasmid Strain Company. The purchased bacterial strain was first inoculated onto solid Luria–Bertani (LB) medium plates and incubated in a biochemical incubator at 25 °C for 1 day to achieve activation. The LB liquid medium consisted of tryptone (10 g/L), yeast extract (5 g/L), and sodium chloride (10 g/L), while the LB solid medium was prepared by adding 20 g/L agar to the liquid medium formulation. After activation, the strain was transferred into LB liquid medium and cultivated for 2 days to obtain a bacterial suspension. The strain was cultivated under aerobic conditions and then introduced into the contaminated soil as an exogenous hydrocarbon-degrading inoculum without further purification; therefore, residual components of the culture medium may also have entered the soil matrix together with the inoculum. In the present study, it was used as a model degrader for diesel-range hydrocarbons rather than for determining its complete physiological optimum or substrate spectrum. The optical density of the suspensions was measured at a wavelength of 600 nm (OD600) using a spectrophotometer (UV-1800, Shimadzu, Kyoto, Japan), and OD_600_ values were used to represent bacterial concentration. When OD_600_ values ranged from 0.2 and 0.8, the following conversion relationship was applied:

(1) $$Y\;=\;8.59\;\times {\;10}^{7}\;\times \;{OD_{600}}^{1.3627}$$

For inoculum preparation, the purchased strain was first activated on solid LB agar plates at 25 °C for 24 h. The activated strain was then transferred into liquid LB medium and incubated at 25 °C for 48 h. The LB medium consisted of 10 g/L tryptone, 5 g/L yeast extract, and 10 g/L NaCl. The resulting 48 h culture suspension was directly used as the inoculum without further washing or separation. Based on Equation (1), the concentration of the inoculum used in this study was approximately 1.15 × 10^8^ cells mL^−1^.

### 2.4. Degradation of Diesel

Diesel hydrocarbons degradation experiments were conducted using four treatment groups: chemical oxidation, the microbial remediation, pre-oxidation combined with microbial remediation, and oxidation combined with microbial remediation. Detailed information is shown in Figure 1b. In the chemical oxidation and microbial remediation groups, contaminated soil was treated exclusively with chemical reagents or microorganisms, respectively. In the pre-oxidation combined with microbial remediation group, the contaminated soil was first subjected to chemical oxidation for 10 days, after which a bacterial suspension was introduced for subsequent microbial remediation. In the oxidation combined with microbial remediation group, oxidants and bacterial suspension were simultaneously added to the soil to achieve diesel removal. The treatment codes were defined as follows: “S” denotes the dosage (%) of SPC^SF^ added to the contaminated soil, and “B” denotes the volume (mL) of the *Bacillus subtilis* suspension added to 100 g of dry soil. For example, S1 and S3 represent treatments with 1% and 3% SPC^SF^ alone, respectively; B10, B20, and B30 represent treatments with 10, 20, and 30 mL bacterial suspension alone, respectively. For combined treatments, codes in the form of B30S1 indicate simultaneous application of 30 mL bacterial suspension and 1% SPC^SF^, whereas codes in the form of S1B30 indicate sequential treatment in which 1% SPC^SF^ was applied first, followed by 30 mL bacterial suspension.

In all treatments involving chemical oxidation, SPC^SF^ was applied at a dosage of 1% of the dry soil weight, while the bacterial suspension was added at levels specified in Table 2. For treatments involving microbial inoculation, the bacterial suspension was directly added to 100 g of diesel-contaminated dry soil and thoroughly mixed to ensure uniform distribution. As shown in Table 2, inoculum volumes of 10, 20, and 30 mL were tested, corresponding to groups B10, B20, and B30, respectively. Based on an inoculum concentration of approximately 1.15 × 10^8^ cells mL^−1^, these addition levels corresponded to initial inoculation amounts of about 1.15 × 10^7^, 2.30 × 10^7^, and 3.45 × 10^7^ cells g^−1^ dry soil, respectively. In the simultaneous coupled treatments, the bacterial suspension and SPC^SF^ were added to the soil at the same time. In the sequential coupled treatments, SPC^SF^ was applied first and the bacterial suspension was introduced in the subsequent stage, as illustrated in Figure 1 and Table 2. This dosage was selected as a moderate and practically relevant level based on previous experience with supported SPC-based oxidation systems and the need to balance oxidation effectiveness with microbial compatibility. The objective of the present study was to evaluate the feasibility and synergistic mechanism of SPC^SF^–microbial coupling under a representative oxidant condition, rather than to optimize the SPC^SF^ dosage over a wide range. Therefore, the 1% dosage should be regarded as a controlled experimental setting rather than a universally optimal value. The term “synergistic” is used in this study in a qualitative sense to indicate that the combined treatment outperformed the individual oxidation or biodegradation treatments. Because no quantitative framework was applied to distinguish additive from truly synergistic behavior, the present results should be interpreted as demonstrating enhanced combined performance rather than rigorously quantified synergism.

In treatments involving chemical oxidation, SPC^SF^ was applied at dosages of 1% or 3% of the dry soil weight, depending on the treatment group, while the bacterial suspension was added at the volumes specified in Table 2. Soil samples from each treatment group were collected on days 5, 10, 17, 24, and 31 for subsequent analyses. All remediation experiments were conducted in triplicate. Independent parallel samples were used for physicochemical analyses.

### 2.5. Analysis Method

Changes in physicochemical properties during the remediation process, which serve as key indicators of remediation progress, were measured and evaluated. ORP and pH were determined using an InLab Redox Electrode and an InLab Routine Pro electrode connected to an FE-28 meter (Mettler-Toledo, Greifensee, Switzerland). For these measurements, a soil-to-water ratio of 1:2.5 was maintained. These parameters were monitored not only to characterize the soil environment, but also to evaluate whether the oxidation conditions created by SPC^SF^ remained compatible with subsequent microbial activity and biodegradation. Measurements were performed using three independent parallel samples, and the results are reported as mean ± standard deviation (SD).

The abundance of diesel hydrocarbon-degrading bacteria in soil was quantified using the plate dilution counting method. Briefly, 1 g of soil sample was transferred into a conical flask containing 99 mL of sterile water and thoroughly homogenized. Serial dilutions were then prepared to obtain microbial suspensions at dilution levels of 10^−3^, 10^−4^, 10^−5^, and 10^−6^ relative to the original sample. Subsequently, 0.1 mL aliquots of each dilution were spread onto LB solid medium plates. The plates were incubated at 25 °C in a constant-temperature biochemical incubator for 24 h, after which colony numbers were counted. Plate counting was used as a cultivation-based indicator of viable hydrocarbon-degrading bacteria under different treatments. However, this method reflects only the culturable fraction of the microbial population and does not provide information on microbial community composition, diversity, or functional gene distribution.

Spectroscopic analyses were conducted to characterize changes in organic matter composition, functional-group characteristics, and surface chemical states during the remediation process, rather than to identify specific transformation products at the compound level. To track carbon transformation during the chemical oxidation and biodegradation processes, dissolved organic matter (DOM) in the contaminated soil was analyzed using 3D-EEM. This analysis identified the formation and evolution of microbially derived and oxidized organic intermediates throughout the remediation. For sample preparation, 5 g of soil was weighed and transferred into a 50 mL centrifuge tube, after which deionized water was added at a soil-to-water ratio of 5:1. The tube was placed in a constant-temperature shaker and agitated at 25 °C and 170 rpm for 24 h. After agitation, the suspension was centrifuged at 3000 rpm for 10 min. The supernatant was then collected and passed through a 0.45 μm membrane filter. The filtrate was stored at 4 °C prior to 3D-EEM analysis. It should be noted that the spectroscopic methods used in this study (3D-EEM, FTIR, and XPS) provide indirect information on compositional and structural changes in organic matter, but do not allow definitive identification of individual transformation products. Therefore, the results were interpreted mainly in terms of molecular transformation features and possible formation of more oxygenated intermediates.

3D-EEM measurements were performed using a Hitachi F-4600 fluorescence spectrophotometer equipped with a 150 W xenon arc lamp (Hitachi, Tokyo, Japan). The excitation wavelength (Ex) was scanned from 200 to 400 nm, and the emission wavelength (Em) was scanned from 250 nm to 550 nm. The wavelength increments were set at 5 nm for excitation and 2 nm for emission, with a scan speed of 1200 nm/min. The obtained fluorescence data were processed using MATLAB R2012a to remove Raman and Rayleigh scattering effects.

The physicochemical properties of samples were characterized using XPS and FTIR. The chemical states of surface elements, including C and Fe, were determined using a K-Alpha XPS system (Escalab 250Xi, Thermo Fisher, Waltham, MA, USA). The analysis was conducted with a spot diameter of 500 μm, a pass energy of 30 eV, and a step size of 0.1 eV. All spectra were calibrated by referencing the C 1s peak to a binding energy of 284.8 eV in order to correct for charging effects. Peak fitting of the XPS spectra was performed using Avantage software (Version 6.6.0). XPS was used to track Fe redox-state changes in the different treatment systems and to assess their possible association with microbial activity.

Fourier transform infrared spectroscopy was carried out using a Nicolet 380 spectrometer (Thermo Fisher, Waltham, MA, USA) to characterize surface functional groups in the soil samples. The soil was passed through a 0.075 mm sieve, and the obtained powder was homogenized with potassium bromide (KBr) to prepare pellets for analysis. For FTIR analysis, 1 mg of dried soil sample was thoroughly mixed and ground with 150 mg of spectroscopic-grade KBr powder. The homogenized mixture was pressed into a circular pellet using a tablet press and then placed in the sample chamber for spectral scanning. Spectra were collected at a resolution of 4 cm^−1^ over a wavenumber range of 400–4000 cm^−1^. FTIR was applied as a supportive technique to characterize changes in aliphatic hydrocarbon-related functional groups and the emergence of oxygen-containing groups during the remediation process. These spectral changes were used as indirect evidence of molecular transformation rather than as a stand-alone proof of biodegradation. Accordingly, the molecular interpretation in this study focused mainly on the transformation of the aliphatic hydrocarbon fraction. Aromatic hydrocarbons present in diesel were not individually quantified or tracked using compound-specific methods; therefore, the reported removal and transformation results should be interpreted as reflecting the selected analytical fraction rather than the complete detoxification of diesel-contaminated soil. No community-level microbial analysis (e.g., high-throughput sequencing) was performed in the present study; therefore, microbial responses were interpreted using plate counts together with physicochemical and spectroscopic evidence.

Given the time and resource requirements of 3D-EEM, XPS, and FTIR analyses, these techniques were applied to a subset of treatment groups to evaluate the primary mechanistic differences among standalone oxidation, standalone microbial remediation, and coupled treatments. The samples were selected based on their remediation mode and overall performance rather than to provide an exhaustive characterization of all experimental conditions. Spectroscopic analyses primarily focused on the control contaminated soil alongside selected SPC^SF^-only, bacteria-only, and combined-treatment configurations that exhibited distinct remediation behaviors. The specific samples analyzed are detailed in the Section 3 and corresponding figure captions.

### 2.6. Statistical Analysis

All remediation experiments and physicochemical analyses were conducted with three independent replicates, and the data are presented as mean ± standard deviation (SD). Statistical analyses were performed using SPSS 26.0 (IBM, Armonk, NY, USA). One-way analysis of variance (ANOVA) followed by Tukey’s multiple comparison test was used to evaluate differences among treatment groups at the same sampling time. Differences were considered statistically significant at *p* < 0.05. Error bars in the figures represent standard deviations of replicate measurements. For quantitative figures, different lowercase letters indicate statistically significant differences among treatments at the same sampling time (*p* < 0.05).

## 3. Results and Discussion

### 3.1. Performance of Diesel Removal

**Figure 2:**
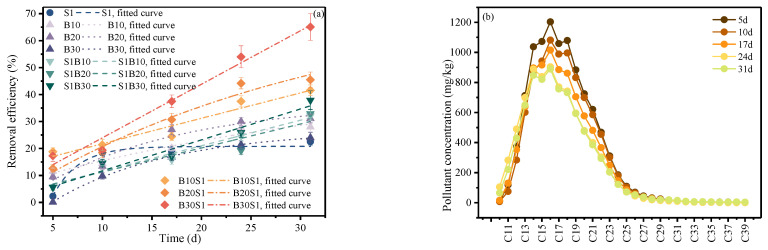
Diesel removal efficiency of different treatment groups (**a**) and concentrations of diesel hydrocarbons with different carbon chain lengths (C10–C40) (**b**). In the group notation, the number “1” in S1 denotes that the dosage of the chemical oxidant was 1% of the dry soil weight; the number “10” in B10 indicates the volume of bacterial suspension added. In S1B10, “1” represents a chemical oxidant dosage of 1% of the dry soil weight, and “10” represents the volume of bacterial suspension added. The numerical designations in other groups follow the same convention. Data are presented as mean ± SD (*n* = 3). Error bars represent standard deviations.

Figure 2 presents the removal efficiencies of different treatment groups. The simultaneous application of chemical oxidation and microbial remediation exhibited the highest removal performance among all treatments, with the B30S1 group achieving a final removal efficiency of 65.1%. This value was significantly higher than those of the chemical oxidation alone and microbial remediation alone groups at day 31 (*p* < 0.05). In contrast, chemical oxidation alone resulted in a removal efficiency of only 22.5% at 31 d, which was lower than that observed for all other treatment groups. This result is consistent with previous studies showing that standalone chemical oxidation can rapidly transform diesel hydrocarbons but often suffers from limited overall efficiency due to inefficient oxidant utilization, radical quenching, and restricted accessibility of strongly sorbed contaminants in soil matrices [33].

The microbial remediation group and the pre-oxidation combined with microbial remediation group showed generally comparable overall diesel removal efficiencies. However, by day 31, the pre-oxidation combined treatment showed a significantly higher removal efficiency than the microbial-only group under the corresponding inoculum condition (*p* < 0.05). In the microbial-only treatment, the removal efficiency initially increased and subsequently decreased with increasing bacterial suspension volume. The maximum removal efficiency in this group was 31.1% at 31 d with a bacterial suspension volume of 20 mL. In contrast, following pre-oxidation, diesel degradation increased consistently with increasing bacterial suspension volume, reaching a final removal efficiency of 37.9%. These observations align with previous reports indicating that moderate pre-oxidation can improve biodegradation by converting recalcitrant hydrocarbons into smaller and more bioavailable molecules, although excessive oxidation may deplete substrates beneficial for sustained microbial growth or generate unfavorable environmental conditions [8,34,35].

In the microbial-only group, limited nutrient availability induced competition among microorganisms, which constrained metabolic activity and resulted in relatively lower removal efficiency. After pre-oxidation, alkane compounds were transformed into smaller carbon molecules that served as accessible nutrients for *Bacillus subtilis*. As a result, the difference in removal efficiency between the microbial-only and pre-oxidation groups gradually decreased after the bacterial suspension was introduced on day 10. By day 31, the pre-oxidation combined with microbial remediation group consistently exhibited higher removal efficiency than the microbial-only group. The simultaneous SPC^SF^–microbial treatment outperformed the sequential mode, suggesting that the supported SPC system not only supplied oxidation-derived biodegradable intermediates but also maintained a physicochemical environment presumably more favorable for concurrent microbial transformation. This finding extends previous reports on oxidation–biological coupling by demonstrating the advantage of a supported sodium percarbonate system in synchronizing oxidation and biodegradation [36,37].

Chemical oxidation was conducted using supported sodium percarbonate as the oxidizing agent and supported ferrous sulfate as the catalytic material. The composition of the supported sodium percarbonate and supported ferrous sulfate are summarized in Figure 1a. It should be noted, however, that the removal efficiencies reported here were obtained in a simplified kaolin system and may be lower in natural soils where oxidant consumption by background organic matter and restricted mass transfer can limit remediation performance.

In addition, the oxidation performance reported in this study was obtained at a single SPC^SF^ dosage of 1% (*w*/*w*, dry soil basis). This dosage was sufficient to produce a clear oxidation effect while maintaining compatibility with microbial remediation in the present model system. However, because the oxidant level was not systematically varied, the current results do not imply that 1% SPCSF showed the most favorable overall remediation performance and compatibility with microbial treatment. In practical applications, the required SPC^SF^ input will likely depend on pollutant concentration, soil composition, oxidant demand, and the tolerance of the associated microbial community.

From an engineering perspective, dosage selection involves a trade-off among remediation efficiency, treatment cost, and environmental disturbance. Lower SPC^SF^ dosages reduce reagent consumption and minimize perturbation to the soil redox environment, but they may fail to generate sufficient reactive species for effective contaminant transformation. Conversely, excessive dosages increase material costs, promote non-productive decomposition pathways, and potentially suppress microbial activity. Therefore, systematic dosage optimization remains an essential step before the scale-up or field application of the SPC^SF^–microbial system.

Compared with previous studies that mainly focused on either oxidant-only treatment or loosely coupled oxidation–biological remediation [38,39], the present results highlight two distinctive features of the SPC^SF^–bacteria system: first, the supported oxidant system maintained sufficient oxidation performance without causing severe incompatibility with bacterial survival; second, the simultaneous treatment mode outperformed the delayed-inoculation mode under the selected conditions. These findings provide a more application-oriented comparison of coupling strategies and therefore strengthen the practical significance of the present study.

The superior performance of the simultaneous coupled treatment suggests that, under the conditions of this study, SPC^SF^ oxidation and microbial degradation were sufficiently compatible to operate synergistically within the same remediation stage. A likely explanation is that the supported SPC^SF^ system provided moderate pollutant activation without imposing an excessively harsh oxidative stress on the bacterial population, thereby allowing oxidation-driven increases in hydrocarbon accessibility and microbial utilization of transformed substrates to proceed concurrently. In contrast, standalone biodegradation was more limited by hydrocarbon hydrophobicity and bioavailability, whereas higher oxidant dosage or less balanced treatment combinations may have imposed less favorable physicochemical conditions for sustained microbial activity. These results indicate that remediation efficiency depended not only on the presence of both oxidation and biodegradation processes, but also on the balance between chemical reactivity and microbial compatibility.

### 3.2. Physicochemical Environment Regulation

#### 3.2.1. pH

**Figure 3:**
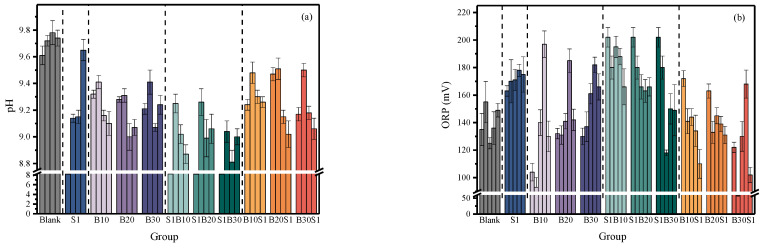
Changes in soil physicochemical properties during remediation: (**a**) pH, (**b**) oxidation–reduction potential (ORP). In the group notation, the number “1” in S1 denotes that the dosage of the chemical oxidant was 1% of the dry soil weight; the number “10” in B10 indicates the volume of bacterial suspension added. In S1B10, “1” represents a chemical oxidant dosage of 1% of the dry soil weight, and “10” represents the volume of bacterial suspension added. The numerical designations in other groups follow the same convention. Data are presented as mean ± SD (*n* = 3). Error bars represent standard deviations. The dashed lines in the figure serve only as separators between different groups.

Soil pH plays a critical role in regulating microbial growth and activity during the remediation process. The pH evolution discussed here reflects the response of the commercial kaolin-based mineral matrix used in this study. Because kaolin has much lower organic buffering complexity than natural organic-rich soils, the magnitude and rate of pH regulation observed here may differ from those expected in field soils containing greater organic matter, more diverse mineral phases, and stronger ion-exchange capacity. As shown in Figure 3a, the pH of the virgin soil was approximately 9.70, indicating strong alkalinity that is unfavorable for microbial survival. Compared with the blank group, the pH of the SPC^SF^-amended group (S1) was reduced. The difference was statistically significant at the initial stage of remediation (*p* < 0.05), confirming the acidifying effect of citric acid present in SPC^SF^. This decrease was primarily attributed to the acidifying effect of citric acid present in SPC^SF^, which effectively lowered soil alkalinity and thereby created more favorable conditions for culturable bacterial survival and associated biodegradation processes. In group S1, a gradual increase in pH with curing time was observed, which can be ascribed to the neutralization effect of alkaline sodium carbonate generated during the decomposition of sodium percarbonate. The relatively high initial pH observed here should be interpreted in the context of the selected model soil. It primarily reflected the intrinsic alkalinity of the commercial kaolin rather than the presence of diesel contamination itself. This alkaline matrix was not intended to represent the most typical pH condition of all oil-contaminated soils; rather, it was used as a controlled and challenging scenario to evaluate whether SPC^SF^ could regulate an unfavorable alkaline environment and thereby improve compatibility with microbial remediation.

For *Bacillus subtilis* used as an aerobic hydrocarbon degrader, neutral to mildly alkaline conditions are generally more favorable for growth and biodegradation than strongly alkaline conditions. Therefore, the initial pH of approximately 9.70 in the present model system should be regarded as a relatively unfavorable starting condition rather than an optimal one for microbial remediation. In this context, the pH-lowering effect of SPC^SF^ was important because it shifted the soil environment toward a range more compatible with bacterial survival and hydrocarbon transformation.

In many field-contaminated soils, pH may be closer to neutral or vary depending on mineralogy, salinity, weathering status, and co-contaminants; therefore, the trends observed in the present study should be understood as mechanistic responses under an alkaline model condition.

During microbial remediation, microorganisms catabolized amino acids from the LB medium to sustain metabolic activity and meet nutrient demands. This process was accompanied by the release of NH_4_^+^, resulting in an increase in soil pH. As nutrients in the LB medium were progressively depleted, microorganisms shifted their metabolic pathways to utilize diesel as carbon sources. The subsequent degradation of these diesel hydrocarbons led to the production of organic acids and the release of H^+^, ultimately causing a decrease in soil pH.

Similarly, in the SB groups with delayed bacterial inoculation, pH values increased following the addition of the bacterial suspension on day 10. This initial increase can be attributed to the continued accumulation of carbonate derived from sodium percarbonate decomposition. Subsequently, as biodegradation-associated microbial processes likely intensified, the generation of organic acids resulted in a gradual decline in soil pH. Accordingly, higher bacterial inoculation levels led to a more pronounced reduction in pH. This trend was statistically significant in the SB treatments at the corresponding sampling stage (*p* < 0.05).

The pH evolution observed in this study aligns with previous reports showing that microbial remediation of diesel hydrocarbons is highly sensitive to soil alkalinity and that suitable pH regulation is essential for maintaining biodegradation activity [40,41]. In many Fenton or Fenton-like oxidation systems, strong acidification or localized oxidant accumulation may inhibit indigenous or inoculated microorganisms [10,42]. In contrast, the SPC^SF^ system moderated the initial alkaline conditions of the contaminated kaolin soil without causing an excessively harsh environment, which may explain its improved compatibility with *Bacillus subtilis*. This highlights an important advantage of the supported SPC-based system over more aggressive oxidant systems.

The combined remediation groups (BS) exhibited a pH variation pattern similar to that observed in the SB group. On day 10, the pH values of groups S1B10, S1B20, and S1B30 were 9.24, 9.47, and 9.17, respectively, which were higher than that of group S1 (8.42). This difference can be attributed to microbial activity that likely consumed citric acid derived from SPC^SF^, thereby weakening its acidifying effect and resulting in relatively higher pH values during the early stage of remediation.

While previous studies have recognized pH as a critical factor controlling hydrocarbon biodegradation [43,44], the present study further demonstrates that a supported SPC-based oxidation system can actively regulate an initially unfavorable alkaline soil environment and thereby improve compatibility with bacterial remediation. This provides a more direct link between oxidant design and microbial environmental adaptation than is commonly addressed in conventional oxidation studies.

#### 3.2.2. ORP

ORP reflects the macroscopic oxidation redox conditions within soil systems. Similarly, the ORP trends observed in this study should be interpreted as the redox response of a simplified mineral substrate rather than that of a natural soil with substantial organic buffering and heterogeneous redox-active phases. In organic-rich soils, stronger buffering, more abundant electron-donating/accepting constituents, and additional radical-scavenging reactions may alter both the absolute ORP values and their temporal evolution during remediation. As shown in Figure 3b, compared with the blank control group, the S1 group exhibited consistently higher ORP from 5 d to 31 d. The increase was statistically significant during the early remediation stage (*p* < 0.05), indicating that SPC^SF^ generated a stronger oxidizing environment in soil. This increase was mainly attributed to the addition of SPC^SF^, which is expected to generate reactive oxidizing species in Fenton-like systems, thereby creating a more oxidative environment in the soil and promoting the degradation of diesel hydrocarbons contaminants.

In the B groups, the ORP values of groups B10, B20, and B30 showed a slow overall increase during the 31 d remediation period. During the first 10 d, the ORP value were slightly lower than those of the blank control group and then gradually declined. During the remediation of diesel-contaminated soil by *Bacillus subtilis*, aerobic respiration represents the dominant metabolic pathway. In this process, diesel hydrocarbons, serving as both carbon and energy sources, are progressively oxidized and decomposed. The electrons (e−) and protons (H+) generated are transferred through a series of electron carriers, such as NADH, FADH_2_, to the terminal electron acceptor, oxygen (O_2_), resulting in the formation of water (H_2_O). This respiratory activity leads to the accumulation of reduced coenzymes and electron carriers, thereby causing a gradual decrease in ORP with increasing remediation time and microbial abundance.

During the pre-oxidation stage, sodium percarbonate oxidized diesel hydrocarbons into small-molecular carbon compounds, which subsequently served as carbon sources for microbial metabolism and facilitated the formation of reduced coenzymes. Consequently, the overall decline in ORP was more pronounced than that observed in the B groups. In the BS groups, sodium percarbonate dominated the initial remediation stage. As the oxidizing agent was gradually consumed, microorganisms increasingly contributed to diesel degradation. As a result, ORP values remained relatively high at the early stage and then gradually decreased throughout the remediation period.

The observed ORP variation patterns align with previous studies indicating that coupling oxidation with bioremediation requires a dynamic balance between oxidative activation and microbial adaptation [45,46,47]. While excessively high ORP can suppress microbial metabolism, a gradual decline following initial oxidation typically indicates increasing microbial participation in contaminant transformation. In this study, the sustained yet moderate ORP levels in the combined treatment groups suggest that SPC^SF^ provided a more controllable oxidative environment than conventional oxidants, which supported the preservation of biological activity during remediation.

The relatively moderate pH and ORP changes observed at the selected 1% SPC^SF^ dosage suggest that this dosage avoided excessively severe physicochemical disturbance in the model soil, although the environmental safety of higher dosages remains to be evaluated in future studies. It should be emphasized that the 1% SPCSF dosage identified in this study should be interpreted only as the most favorable condition within the tested experimental range, rather than as a universally optimized dosage. The appropriate oxidant dosage is expected to depend on site-specific factors such as soil composition, contaminant load, natural organic matter content, and microbial tolerance.

In contrast to studies that interpret ORP primarily as an oxidation indicator [48,49], the present work uses ORP together with cultivation-based bacterial abundance and spectroscopic evidence to show how redox regulation is linked to biodegradation-related processes in the coupled system. This combined interpretation enhances the mechanistic significance of the ORP results.

It should be noted that pH and ORP in this study were used as bulk physicochemical indicators of changes in the remediation environment. Although these parameters are informative for evaluating overall system conditions, they should not be interpreted as direct proxies for specific biochemical pathways, Fe redox reactions, or reactive oxygen species dynamics. Therefore, mechanistic interpretation based on pH and ORP remains indirect and should be considered together with other lines of evidence.

#### 3.2.3. Abundance of Culturable Hydrocarbon-Degrading Bacteria

**Figure 4:**
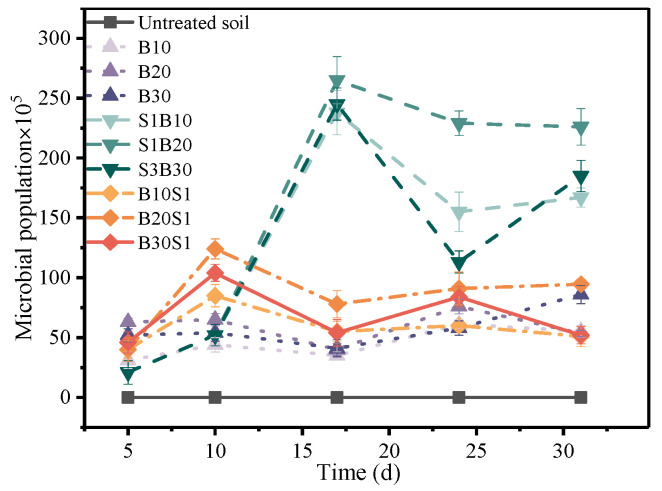
Temporal variation in the abundance of culturable hydrocarbon-degrading bacteria under different remediation treatments. The blank control represents contaminated soil treated with sterile solution only. B10, B20, and B30 denote bacteria-only treatments with different inoculum volumes; B10S1, B20S1, and B30S1 denote simultaneous SPC^SF^–bacteria treatments; and S1B10, S1B20, and S1B30 denote delayed-inoculation treatments in which bacterial suspension was added after 10 d of SPC^SF^ treatment. Data are presented as mean ± SD (*n* = 3). Error bars represent standard deviations. Plate counts reflect the abundance of culturable hydrocarbon-degrading bacteria and do not represent the full microbial community.

As shown in Figure 4, plate counting was used to evaluate the temporal dynamics of culturable hydrocarbon-degrading bacteria across nine treatment groups, in order to determine whether the inoculated bacteria survived and proliferated in the substrate during remediation. Contaminated soil treated with sterile solution served as the blank control, in which no detectable colonies were observed throughout the experimental period. Importantly, culturable bacteria remained detectable in the inoculated treatments throughout the 31-day remediation period, indicating that the introduced strain was able to survive in the substrate under the tested conditions.

In Group B (B10, B20, and B30), which received only the *Bacillus subtilis* ATCC 11774 inoculum, the abundance curves at different inoculation dosages exhibited fluctuating patterns of alternating growth and decline over the 31-day remediation period. This fluctuation likely reflects the dynamic adaptation of the strain to the soil environment and the gradual shift in available carbon sources during remediation. Within the first 24 days, the B20 group showed the most sustained increase in culturable bacterial abundance, which corresponded well with its relatively higher diesel degradation efficiency shown in Figure 2. The delayed increase in the B10 group can be attributed to its lower initial inoculum density, which required more time to establish a comparable culturable population. In contrast, the high initial bacterial input in the B30 group may have intensified competition for limited nutrients, leading to faster nutrient depletion and less stable population maintenance.

In the simultaneous composite treatments containing both SPC^SF^ and the bacterial inoculum (B10S1, B20S1, and B30S1), the B20S1 group exhibited the highest abundance of culturable bacteria, which was consistent with the dosage-dependent trend observed in Group B. Moreover, at the same inoculation level, the abundance of culturable bacteria in the simultaneous SPC^SF^–bacteria treatments was generally higher than that in the corresponding bacteria-only groups (i.e., B10S1 > B10, B20S1 > B20, and B30S1 > B30). This result suggests that, under the selected SPC^SF^ dosage, the supported oxidation system created conditions more favorable for bacterial persistence and proliferation than microbial treatment alone. Compared with their corresponding initial levels, the abundance of culturable bacteria in the coupled treatments generally increased during the main remediation stage, although the magnitude and stability of the increase depended on inoculum level and treatment mode.

For the delayed-inoculation SB group (in which bacterial suspension was introduced on day 10 after prior SPC^SF^ treatment), a small number of colonies was detected on day 5 before external bacterial inoculation. Because plate counting reflects only the culturable fraction of the microbial population, this observation should be interpreted cautiously; it may indicate the presence of a low background culturable population and/or minor contamination introduced during handling, rather than definitive activation of a specific indigenous degrading community. After bacterial inoculation on day 10, the abundance of culturable bacteria in the SB group increased more rapidly than that in the simultaneous-addition treatments, indicating that prior SPC^SF^ treatment may have improved substrate availability and physicochemical conditions for subsequent bacterial establishment.

Overall, the plate count results are consistent with the trends observed for pH regulation, ORP evolution, and diesel removal efficiency. In particular, the relatively higher abundance of culturable bacteria in the combined treatments suggests that SPC^SF^ did not impose severe inhibition under the selected dosage, but instead may have promoted the formation of a more favorable physicochemical environment and more accessible carbon substrates for bacterial persistence. These cultivation-based results support the interpretation that the enhanced degradation efficiency in the coupled treatments was associated with both oxidation-derived substrate activation and bacterial transformation. Therefore, although plate counting does not provide community-level resolution, the observed abundance dynamics do substantiate the microbiological nature of the coupled remediation process at the cultivation-based level. However, because plate counting captures only the culturable fraction of the microbial population, these results should be regarded as supportive rather than comprehensive evidence of microbial response. No conclusion regarding microbial community structure, diversity, succession, or functional gene distribution can be drawn from this analysis alone.

Together with the pH and ORP results, the cultivation-based abundance data suggest that the coupled SPC^SF^–bacteria treatments provided a more favorable environment for biodegradation-related processes than the standalone treatments. To further elucidate how this enhancement occurred at the molecular and redox scales, the transformation of dissolved organic matter, carbon functional groups, and iron speciation was subsequently investigated.

These abundance patterns are consistent with previous reports showing that the success of hydrocarbon bioremediation depends strongly on whether chemical pretreatment alleviates environmental stress while simultaneously generating more bioavailable carbon substrates [50,51]. In this respect, the present study adds cultivation-based evidence that the supported SPC^SF^ system not only transformed diesel hydrocarbons chemically, but also promoted conditions favorable for bacterial persistence in the substrate.

The inoculated bacterial suspension may have introduced residual medium-derived organic components, which could have contributed not only to bacterial establishment but also to redox-related processes in the coupled system.

Taken together, the pH, ORP, and bacterial count results indicate that the effectiveness of the coupled remediation system depended strongly on the dynamic regulation of the soil microenvironment. Oxidation created a transiently more oxidative and chemically reactive condition, which could improve hydrocarbon activation, while the supported SPCSF system appeared to moderate this perturbation to a level still compatible with bacterial survival and activity under the optimal simultaneous treatment. This balance between pollutant activation and microbial tolerance is likely one of the key reasons for the superior performance of the simultaneous coupled treatment observed in this study.

### 3.3. Mechanism of Enhanced Remediation

In the present study, *Bacillus subtilis* was treated as an aerobic diesel-degrading in-oculum in a model soil system, and the mechanistic discussion focuses on its functional participation in coupled remediation rather than on determining its full physiological growth range. To elucidate the synergistic mechanism responsible for the enhanced remediation performance of the SPC^SF^–microbial system, the transformation of organic carbon, regulation of soil redox conditions, and iron speciation were systematically investigated using spectroscopic and electrochemical techniques. The mechanistic interpretation proposed in this section is based on integrated evidence from remediation performance, physicochemical parameters, and spectroscopic characterization To highlight the dominant transformation pathways without introducing excessive redundancy, the advanced spectroscopic analyses in this section were conducted on representative treatment groups selected from the overall experimental matrix. Microbial responses were evaluated primarily through cultivation-based abundance measurements and indirect transformation signals, rather than through community-level analysis. Accordingly, the mechanistic interpretation does not extend to microbial community structure, diversity, or functional gene-level responses. In this context, “organic carbon transformation” specifically refers to the breakdown of aliphatic hydrocarbon structures and the concurrent evolution of oxygen-containing functional groups during the coupled oxidation–biodegradation process. Specifically, 3D-EEM, FTIR, and XPS were employed to characterize biodegradation-related transformations through the evolution of dissolved organic matter, the conversion of carbon functional groups, and variations in Fe/C chemical states. Together, these analyses provided molecular-scale evidence of intermediate formation and microbial conversion pathways, allowing biodegradation to be evaluated beyond overall diesel removal efficiency.

#### 3.3.1. Transformation of Diesel Hydrocarbons and DOM Evolution

**Figure 5:**
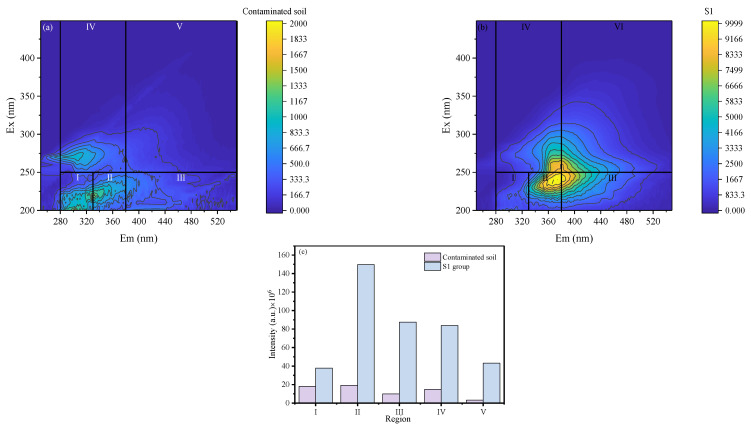
Three-dimensional fluorescence excitation-emission matrix (3D-EEM) spectra of (**a**) the original contaminated soil and (**b**) the S1 group, and (**c**) integrated fluorescence volumes of each region. S1 was selected as the representative SPC^SF^ treatment group because it exhibited the oxidation products of diesel among the oxidation treatments. The fluorescence signals were classified into five regions: I (aromatic protein I), II (aromatic protein II), III (fulvic-like acids), IV (soluble microbial by-products), and V (humic-like ac-ids).

Changes in soil organic matter composition reflect both the transformation of diesel hydrocarbons and the growth and metabolic activity of microorganisms. To represent the coupled remediation system with the highest removal performance, 3D-EEM analysis was conducted on the original contaminated soil and the representative group S1 (SPC^SF^ only). 3D-EEM was employed to track changes in the composition of dissolved organic matter during biodegradation. As shown in Figure 5a,b, the fluorescence signals were classified into five regions: I (aromatic protein I), II (aromatic protein II), III (fulvic-like acids), IV (soluble microbial by-products), and V (humic-like acids). Figure 5a presents the 3D-EEM spectra of the original contaminated soil, in which region II dominated the fluorescence response. After remediation, additional fluorescence signals appeared in regions III, IV, and V, indicating the formation of fulvic-like and protein-like substances. These changes are consistent with the generation of oxidation-derived intermediates and their subsequent transformation under microbially influenced conditions.

These components originated from the oxidative cleavage of C–C and C–H bonds in long-chain diesel hydrocarbons, followed by the presumed microbial utilization and transformation of the resulting low-molecular-weight intermediates. The marked increase in fluorescence intensity within regions IV and V indicates the formation of soluble microbial by-products and humified organic substances and suggests that diesel removal in the coupled system involved biodegradation-related transformation in addition to abiotic oxidation. These intermediates were subsequently assimilated by microorganisms for biomass synthesis and organic matter production, thereby enhancing DOM abundance and fluorescence intensities in regions III, IV, and V.

The integrated fluorescence volumes of each region are shown in Figure 5c. For the original contaminated soil, the integrated volumes of regions I–V were 17,986,163.25, 19,177,989.8, 9,974,061.3, 14,780,727.44, and 3,159,094.84 au·nm^2^, respectively. In contrast, the corresponding values in the BS group increased markedly to 37,842,111.38, 149,662,898.34, 87,489,665.46, 83,846,487.92, and 43,058,974.35 au·nm^2^, respectively. The substantial increase in regions associated with microbial by-products and humic/fulvic-like substances indicates that diesel removal in the coupled system involved active biodegradation and subsequent organic matter transformation, rather than solely abiotic oxidation. The fluorescence intensities of humic-like and fulvic-like substances increased to 8.77 and 13.63 times those of the original contaminated soil, respectively. Overall, SPC^SF^ promoted the oxidation and fragmentation of diesel hydrocarbons into bioavailable substrates, which supported microbial metabolism and facilitated the production of organic acids.

Spectroscopic results are consistent with observations that chemical oxidation fragments diesel hydrocarbons into lower-molecular-weight compounds, which are subsequently incorporated into microbial metabolism and dissolved organic matter pools [9,32]. The increase in fulvic-like, humic-like, and soluble microbial by-product signals in the BS group suggests that the supported oxidation system promotes a transformation pathway distinct from simple abiotic oxidation, characterized by the active microbial reprocessing of oxidation-derived intermediates.

#### 3.3.2. Microbial Involvement in Fe(II) Stabilization and Fenton Cycling

**Figure 6:**
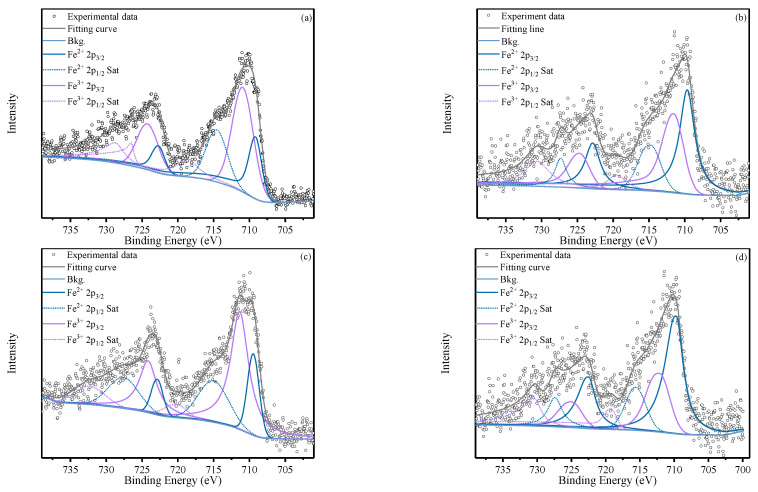
X-ray photoelectron spectroscopy (XPS) Fe 2p spectra of soil samples. (**a**) S1, (**b**) B30S1, (**c**) S3 and (**d**) B30S3.

For Fe speciation analysis, representative groups were selected to compare oxidation alone (S1, S3) and coupled treatments under different oxidant dosages (B30S1 and B30S3). This selection allowed the influence of SPC^SF^ dosage on Fe redox-state evolution to be compared directly. XPS analysis was conducted to further elucidate iron speciation in SPC^SF^ combined with bacterial suspension. The XPS results revealed differences in the Fe redox state between the SPC^SF^-only and SPC^SF^–bacteria systems. This, along with variations in ORP and observed remediation behavior, suggests the potential involvement of microbial activity in Fe redox transformations. However, because the bacterial suspension also introduced residual medium components and because no direct Fe(III)-reduction assay was performed, the observed Fe redox changes cannot be attributed exclusively to bacterial action. As shown in Figure 6, the Fe 2p spectra of both the S group (SPC^SF^ alone) and the SB groups (SPC^SF^ combined with bacterial suspension) exhibited characteristic Fe 2p_3/2_ and Fe 2p_1/2_ peaks, along with associated satellite peaks arising from energy loss during photoemission. The Fe^2+^ 2p_3/2_ and 2p_1/2_ peaks were located at approximately 709.0 eV and 722.0 eV, whereas the corresponding Fe^3+^ peaks appeared near 711.0 eV and 724.0 eV.

In the S groups, the Fe 2p spectra were dominated by Fe^3+^ signals. Quantitative peak deconvolution yielded Fe^2+^/Fe^3+^ ratios of 36.80%/63.20% in group S1 and 26.65%/73.35% in group S, indicating that iron predominantly existed in the Fe(III) state.

In contrast, the SB groups, particularly B30S1 and B30S3, exhibited substantially enhanced Fe^2+^ peak intensities. The Fe^2+^/Fe^3+^ ratio increased to 53.38%/46.62% in B30S1 and further to 66.73%/33.27% in B30S3, corresponding to Fe^2+^ increases of 16.58% and 40.08% relative to S1 and S3, respectively. The observed enrichment of Fe^2+^ indicates that the presence of microorganisms was associated with enhanced Fe(II) accumulation in the coupled system. A plausible explanation is that bacterial growth and metabolism, as well as metabolites released during remediation, altered the local redox microenvironment and thereby helped maintain Fe in a more reduced and reactive state. In addition, residual organic components introduced with the bacterial suspension medium may also have contributed to Fe redox-state regulation and should not be excluded as a possible influencing factor.

The Fenton reaction is governed by the cyclic redox transformation between Fe(II) and Fe(III), which sustains the continuous generation of hydroxyl radicals (·OH) for the oxidative degradation of diesel hydrocarbons. Among the controlling factors, Fe(II) concentration is crucial for catalyzing hydrogen peroxide (H_2_O_2_) to produce ·OH. If microbial activity contributed to maintaining Fe(II) availability, it may have supported continued Fenton-like reactivity and enhanced contaminant degradation.

Moreover, because Fe(III) is prone to precipitation and deactivation under alkaline conditions, maintaining a high proportion of Fe(II) not only reduces total iron consumption but also supports the sustained and efficient operation of the Fenton reaction system. Supported by ORP and XPS results, *Bacillus subtilis* ATCC 11774 is hypothesized to drive this increased Fe(II) availability in the coupled system; however, the specific Fe(III)-to-Fe(II) reduction pathway was not directly verified.

This interpretation is supported by previous studies reporting that microorganisms influence iron speciation, thereby affecting radical generation efficiency in Fenton-like systems [52,53,54]. Maintaining Fe(II) availability is a critical factor controlling hydroxyl radical production and oxidant utilization efficiency [55,56]. Under this hypothesis, the bacterial contribution would be indirect rather than necessarily enzymatic, involving redox-active metabolites, oxygen consumption, local electron-donor generation, and/or microenvironmental shifts that slow Fe(II) oxidation or promote Fe(III) re-reduction. While earlier studies primarily focused on Fe-activated SPC in aqueous systems, the present results suggest that microbial participation in soil matrices may help maintain a higher Fe(II) proportion and thus favor continued Fe(II)/Fe(III) redox cycling. At the same time, this effect should be regarded as a working hypothesis rather than a directly demonstrated pathway in the present study. A plausible mechanism is that bacterial metabolism, microbial metabolites, and associated redox microenvironment changes indirectly supported Fe(II) persistence. In addition, residual components of the bacterial suspension medium may also have influenced Fe redox behavior and cannot be excluded. Therefore, direct Fe(III)-to-Fe(II) reduction by *Bacillus subtilis* remains unverified here and requires targeted follow-up experiments. Because neither reactive oxygen species nor microbial metabolic pathways were directly measured in this study, the proposed coupling among bacterial activity, Fe redox transformation, and sustained oxidizing reactivity should be regarded as an inferred working hypothesis rather than a directly demonstrated mechanism.

#### 3.3.3. Molecular-Scale Evidence for Carbon Structure Transformation

**Figure 7:**
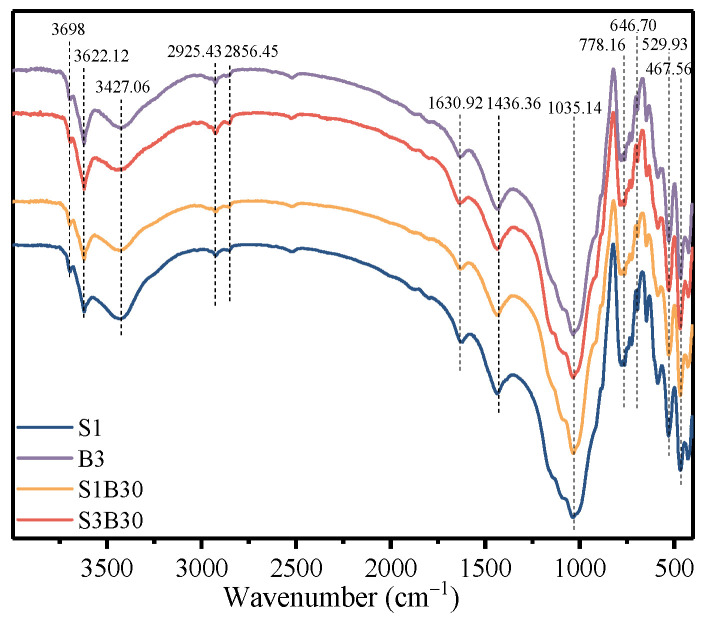
FTIR of soil samples. S1, S1B30, S3 and S3B30.

Similarly, FTIR and XPS C 1s analyses were performed on representative SPC^SF^-only and combined-treatment groups to compare the molecular transformation of carbon structures under distinct remediation modes. FTIR and XPS analyses were conducted to characterize the molecular-level transformation of petroleum-derived carbon during remediation. FTIR tracked changes in hydrocarbon-associated and oxygen-containing functional groups, while XPS quantified variations in carbon bonding environments, such as C–C, C–O–C, and O–C=O. In this context, “carbon structure transformations” refers to the oxidative cleavage of long-chain hydrocarbons and the subsequent formation or consumption of oxygen-containing intermediates and carboxyl-rich products under the coupled action of SPC^SF^ and microorganisms. The FTIR spectra of Groups S (SPC^SF^ only), SB (SPC^SF^ combined with bacteria), and B (bacteria only) are shown in Figure 7. The absorption band at 3622 cm^−1^, attributed to the hydration of clay minerals, exhibited significantly higher intensity in the microbial treatment groups (Groups B and SB) than in Group S. This enhancement reflects increased soil water-binding capacity associated with microbial activity.

Characteristic absorption bands related to soil organic matter were observed at 2925 and 2856 cm^−1^, corresponding to aliphatic C–H stretching vibrations, and at around 1630 cm^−1^, generally attributed to the H–O–H bending vibration of adsorbed water in the soil/mineral matrix. Additional bands at 2960, 2896, 778, and 693 cm^−1^, indicate the presence of residual aliphatic and aromatic hydrocarbon components. A decrease in the relative intensity of aliphatic C-H bands was interpreted as evidence of hydrocarbon chain cleavage and consumption, whereas an increase in oxygen-containing bands, particularly those associated with carboxyl functionalities, was taken to indicate the generation of more oxidized transformation products.

Notably, the Si–O–Si stretching band at 1035 cm^−1^ was significantly more intense in Group SB than in Group S, suggesting enhanced removal of contaminants from clay mineral surfaces and increased exposure of kaolinite structural units. The absorption band at 1436 cm^−1^, associated with carboxylic acid functional groups, also exhibited higher intensity in Group SB, suggesting that microbial participation promoted the further transformation of oxidation-derived intermediates into carboxyl-rich products. The increased abundance of carboxyl-containing structures is consistent with the microbial conversion of partially oxidized hydrocarbons into more polar and biodegradable products.

**Figure 8:**
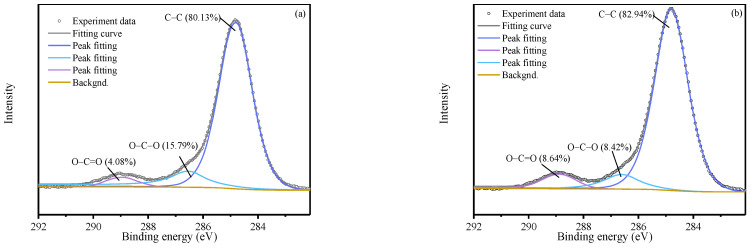

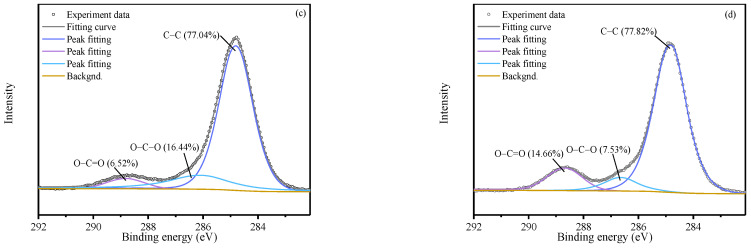
X-ray photoelectron spectroscopy (XPS) C 1s spectra of soil samples. (**a**) S1, (**b**) B30S1, (**c**) S3 and (**d**) B30S3.

XPS C 1s spectra (Figure 8) further supported these transformations. Peaks at binding energies of 284.80 eV, 286.54 eV, and 288.95 eV were assigned to C–C, C–O–C, and O–C=O functional groups, respectively. The C–C component primarily represents reduced hydrocarbon structures, while the C–O–C and O–C=O components correspond to more oxidized carbon species. Shifts in the relative abundance of these components demonstrate the conversion of diesel hydrocarbons into oxygenated intermediates and carboxyl-rich products during remediation. At identical SPC^SF^ dosages, the relative intensity of the O–C=O peak was substantially higher in the SB groups than in the corresponding S groups (e.g., B30S1: 8.64% > S1: 4.08%; B30S3: 14.66% > S3: 6.52%), indicating enhanced conversion of oxidative intermediates into carboxylic acid metabolites.

Conversely, the relative intensity of C–O–C peak was markedly lower in the SB groups (B30S1: 8.42% > S1: 15.79%; B30S3: 7.53% > S3: 16.44%), suggesting active microbial utilization and transformation of epoxide intermediates ether- or epoxide-containing intermediates. The combined FTIR and XPS results indicate that microbial biodegradation converted oxidized hydrocarbon intermediates into more stable, potentially mineralizable carboxyl-rich compounds, providing molecular-scale evidence for the enhanced degradation efficiency observed in the SB treatment.

FTIR and XPS results align with the established transformation pathway in which diesel hydrocarbons are progressively converted from reduced aliphatic structures into oxygenated intermediates and ultimately into polar, carboxyl-rich compounds. While previous studies typically inferred this transformation from pollutant disappearance or bulk oxidation performance [6,12,21], the present study provides direct spectroscopic evidence that microbial participation accelerates the downstream conversion of oxidized intermediates. This is significant because it moves the interpretation of coupled remediation beyond macroscopic removal efficiency toward molecular-scale understanding of transformation pathways.

It should be noted that these spectroscopic analyses were performed on representative groups for mechanistic interpretation and were not intended to provide complete characterization of every treatment combination in the experimental matrix.

### 3.4. Implications and Limitations for Application in Natural Soils

The present study was performed in commercial kaolin, which provided a simplified model matrix for isolating the coupled effects of SPC^SF^ and *Bacillus subtilis*. The present matrix was commercial kaolin, a mineral substrate whose physicochemical behavior differs from that of organic-rich natural soils. In particular, the ion-exchange capacity, buffering behavior, and organic-mineral interactions of kaolin differ substantially from those of soils containing higher levels of humic substances, clay–organic associations, and dissolved organic matter. As a result, the pH and ORP modulation observed in this study may be more direct and less buffered than would be expected in organic-rich field soils. While this design is advantageous for mechanistic interpretation, the remediation behavior in natural soils may differ substantially due to geochemical, biological, and physical complexities. Natural soils generally contain higher levels of dissolved and particulate organic matter, which can compete with target hydrocarbons for reactive oxygen species and act as radical scavengers, thereby decreasing oxidant utilization efficiency. In addition, inorganic constituents such as bicarbonate, carbonate, chloride, sulfate, and phosphate may participate in non-productive side reactions or alter iron speciation, which could weaken Fenton-like reactivity. The initially alkaline pH of the model system was primarily determined by the commercial kaolin matrix and should not be regarded as representative of all diesel-contaminated field soils, many of which exhibit neutral or moderately alkaline conditions. This distinction from many previous laboratory studies is important: rather than directly extrapolating performance to field conditions, the present study positions its contribution primarily in mechanistic clarification under a controlled model matrix.

Beyond these geochemical interferences, the presence of native microbial communities introduces biological outcomes that are more complex than those observed in the present model system. Indigenous microorganisms may compete with the introduced *Bacillus subtilis* for nutrients and ecological niches, potentially suppressing inoculum survival; however, they may also cooperate in the transformation of oxidation-derived intermediates and broaden the biodegradation spectrum. Therefore, the net biological effect in natural soils depends strongly on the composition and adaptability of the resident microbiota.

Physical transport and delivery limitations are also expected to be more pronounced in field soils. Compared with commercial kaolin, natural soils typically exhibit greater pore-scale heterogeneity, stronger contaminant aging, more complex sorption domains, and lower accessibility of both oxidant and bacteria to hydrocarbon-contaminated microsites. These factors reduce contact efficiency among SPC^SF^, iron species, microorganisms, and sorbed diesel hydrocarbons, thereby weakening the apparent synergy observed under laboratory conditions.

Although the current results demonstrate the mechanistic feasibility of SPC^SF^–microbial coupling, they should not be interpreted as a direct prediction of field-scale performance. Future studies should evaluate this system in representative natural soils with varying organic matter contents, ionic compositions, and indigenous microbial communities, while further examining oxidant transport, radical scavenging intensity, and delivery efficiency under realistic environmental conditions.

Another limitation relevant to engineering application is that only one SPC^SF^ dosage was evaluated in the main remediation experiment. Although 1% (*w*/*w*) provided a useful basis for mechanistic comparison under controlled conditions, the optimal dosage in real soils may differ substantially depending on contaminant aging, natural oxidant demand, organic matter content, and delivery efficiency. In field settings, overdosing may increase cost and the risk of unnecessary oxidant consumption or ecological disturbance, whereas underdosing may lead to insufficient hydrocarbon activation. Thus, site-specific dosage optimization will be required for practical deployment. In addition, a further limitation of this study is that microbial evaluation was based mainly on cultivation-dependent plate counting, together with indirect evidence from diesel transformation, pH/ORP evolution, and spectroscopic characterization. While this framework is useful for identifying biodegradation-related trends, it does not capture the full microbial community structure, diversity, or functional succession during remediation. Therefore, the specific taxa involved, their ecological interactions, and the functional pathways responsible for hydrocarbon degradation and possible Fe redox transformation remain unresolved. Future studies should incorporate high-throughput sequencing, quantitative PCR, and/or transcriptomic approaches to more directly characterize microbial community dynamics and function in SPC^SF^–microbial coupled systems.

From a practical perspective, the present results should be interpreted as laboratory-scale mechanistic evidence rather than direct proof of field applicability. During scale-up, several additional factors are likely to influence treatment performance, including spatial heterogeneity of soil properties, contaminant aging, uneven distribution of petroleum hydrocarbons, and limited contact efficiency among SPC^SF^, iron species, microorganisms, and sorbed contaminants. These factors may reduce the apparent synergy observed under controlled laboratory conditions and complicate oxidant and bacterial delivery in field soils.

Cost is another important consideration for engineering implementation. The overall treatment cost would depend on SPC^SF^ dosage, preparation of the supported oxidant and catalyst system, bacterial cultivation and delivery, and the site-specific oxidant demand of contaminated soil. Although the supported SPC^SF^ design may improve oxidant utilization efficiency and reduce unnecessary reagent consumption compared with less controlled systems, a formal techno-economic assessment was beyond the scope of the present study.

In addition, environmental risk should be considered before field deployment. Excessive oxidant input or nonuniform delivery may cause unnecessary disturbance to soil pH and ORP, increase non-productive oxidant consumption, and potentially affect native microbial communities. Therefore, prior to practical application, site-specific evaluation of dosage, delivery method, treatment radius, and ecological compatibility will be necessary to balance remediation efficiency with environmental safety.

When considered together, the spectroscopic results suggest a coordinated transformation pathway during the coupled remediation process. The 3D-EEM results indicate changes in soluble organic matter characteristics and imply the generation or consumption of intermediate fluorescent substances during oxidation and biodegradation. FTIR further suggests the attenuation of aliphatic hydrocarbon-related structures and the emergence of oxygen-containing functional groups, which is consistent with partial oxidation and subsequent biological utilization of more polar intermediates. Meanwhile, XPS reveals changes in the surface chemical states of carbon, oxygen, and iron, supporting the view that hydrocarbon oxidation and Fe redox transformation occurred simultaneously within the supported oxidant system. Therefore, rather than representing isolated observations, these three analytical approaches collectively support a mechanism involving pollutant activation by SPC^SF^, physicochemical environment regulation, and subsequent microbial transformation of oxidized products.

## 4. Conclusions

This study demonstrated that coupling *Bacillus subtilis* with the supported sodium percarbonate composite SPC^SF^ is a feasible strategy for the remediation of diesel-contaminated soil, and that treatment sequence plays a decisive role in remediation effectiveness. Among the tested treatments, the sequential coupled mode showed the best overall performance because pre-oxidation by SPC^SF^ enhanced pollutant accessibility while avoiding the stronger inhibitory effects on microorganisms that may occur under simultaneous oxidation–biodegradation conditions.

The results further indicate that the remediation advantage of the coupled system is not simply due to the superposition of chemical oxidation and biodegradation, but rather to the dynamic regulation of the soil physicochemical environment. Changes in pH, ORP, bacterial abundance, and spectroscopic characteristics collectively suggest that SPC^SF^ promoted the initial transformation of diesel hydrocarbons and modified the redox environment, whereas the subsequent microbial stage contributed to the further conversion of oxidized intermediates under more biologically compatible conditions.

In addition, the SPC^SF^ dosage applied in this study was selected as a representative controlled experimental condition rather than a fully optimized application rate. Although the 1% SPC^SF^ treatment exhibited the best overall performance within the tested range, further dosage optimization will be required before field application under more complex and heterogeneous soil conditions.

From a mechanistic and practical perspective, this work highlights that the compatibility between oxidant-induced activation and microbial recovery is a key factor in designing effective coupled remediation systems. Thus, optimizing oxidant delivery form and treatment sequence may be as important as improving oxidant strength itself.

Nevertheless, this study was conducted in a simplified kaolin-based model matrix, and the analytical evaluation focused mainly on diesel-range aliphatic hydrocarbons and indirect molecular transformation evidence. Therefore, caution is needed when extrapolating the present findings to natural soils and to full ecotoxicological risk reduction. Future studies should incorporate natural soil heterogeneity, compound-specific analysis of aromatic hydrocarbons, and higher-resolution biodegradation assessment methods to further validate and extend the applicability of the SPC^SF^–microbial remediation strategy.

## Figures and Tables

**Table 1:** Physicochemical properties of kaolin and diesel.

Materials	Test Content	Value	Standard/Method
Kaolin	Plastic limit (ω_P_, %)	18.7	ASTM D4318-10 [25]
	Liquid limit (ω_L_, %)	28.3	
	Plasticity index (I_P_, %)	9.6	
	Specific surface area (m^2^/g)	7.98	GB/T 19587-2017 [26]
	Specific gravity	2.77	ASTM D854-14 [27]
	pH	9.23	ASTM D4972-13 [28]
	Sand: 75–2000 μm	5.69	Laser particle size analysis (Mastersizer 3000, Malvern Panalytical, Malvern, UK)
	Silt: 5–75 μm	70.10	
	clay: <5 μm	24.21	
Diesel	Density (25 °C, g/cm^2^)	0.80	GB/T 1884-2000 [29]
	Dynamic viscosity (25 °C, MPa/s)	4.6	GB/T 265-1988 [30]

## Data Availability

The original contributions presented in this study are included in the article. Further inquiries can be directed to the corresponding authors.

## Abbreviations

The following abbreviations are used in this manuscript:

SPC	Sodium percarbonate
SPC^SF^	A silica gel-loaded, iron-catalyzed SPC composite
ORP	oxidation–reduction potential
LB	Luria–Bertani
OD_600_	Optical density of the suspensions at a wavelength of 600 nm
DOM	dissolved organic matter
Ex	excitation wavelength
Em	emission wavelength
KBr	potassium bromide
